# Supplementary material for: Knockout of the neonatal Fc receptor alters immune complex trafficking and lysosomal function in cultured podocytes
Source: PLoS One. 2023 Apr 18;18(4):e0284636. doi: 10.1371/journal.pone.0284636 (PMC10112810; doi:10.1371/journal.pone.0284636)

Figure 4

LAMP1 4h

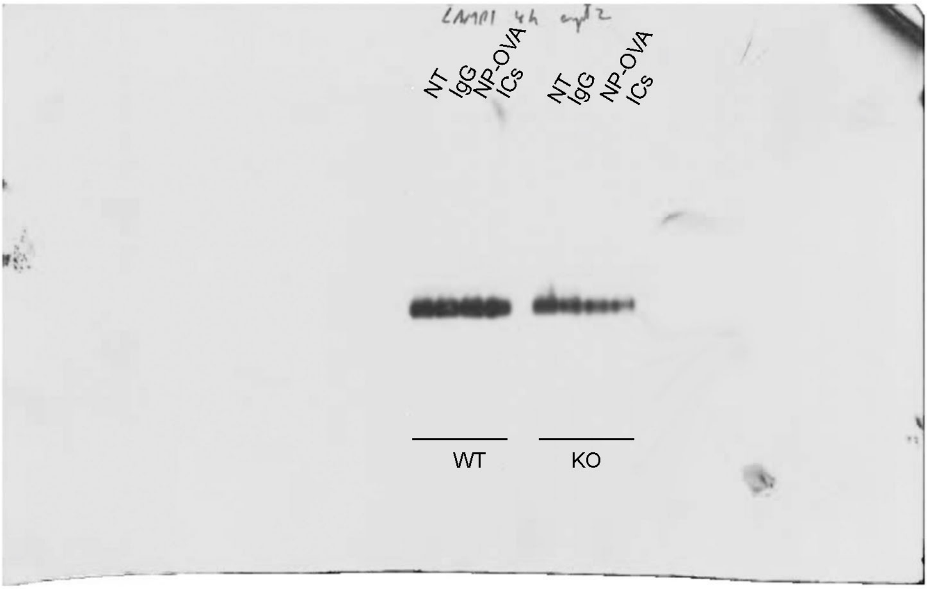

Cathepsin B 4h

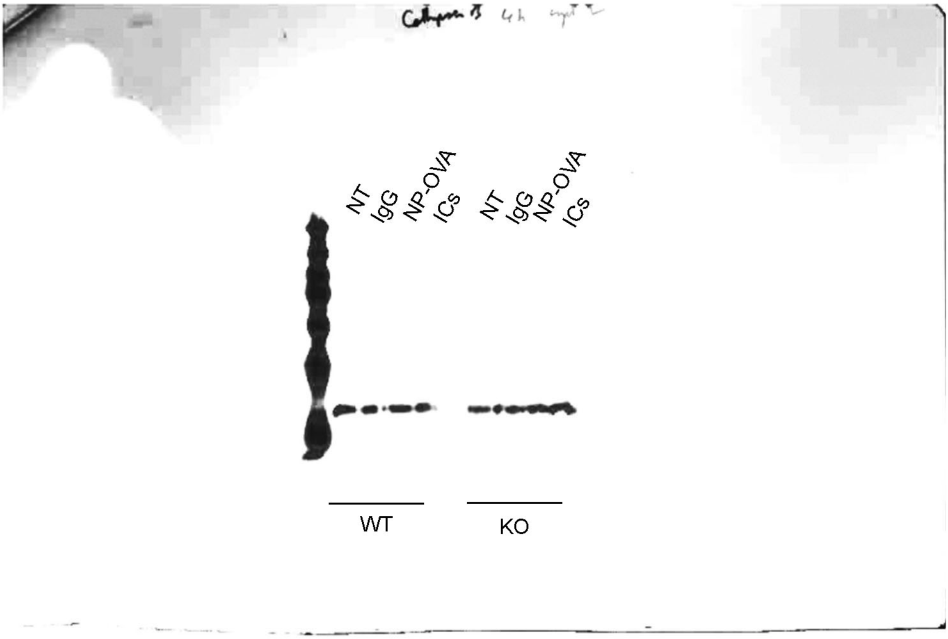

Beta actin 4h

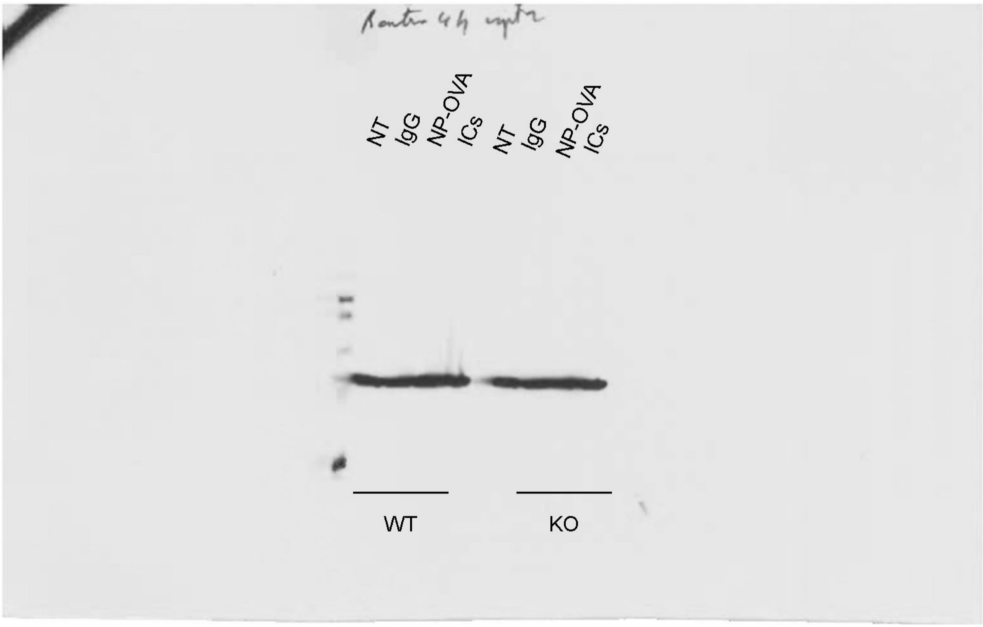

LAMP1 48h

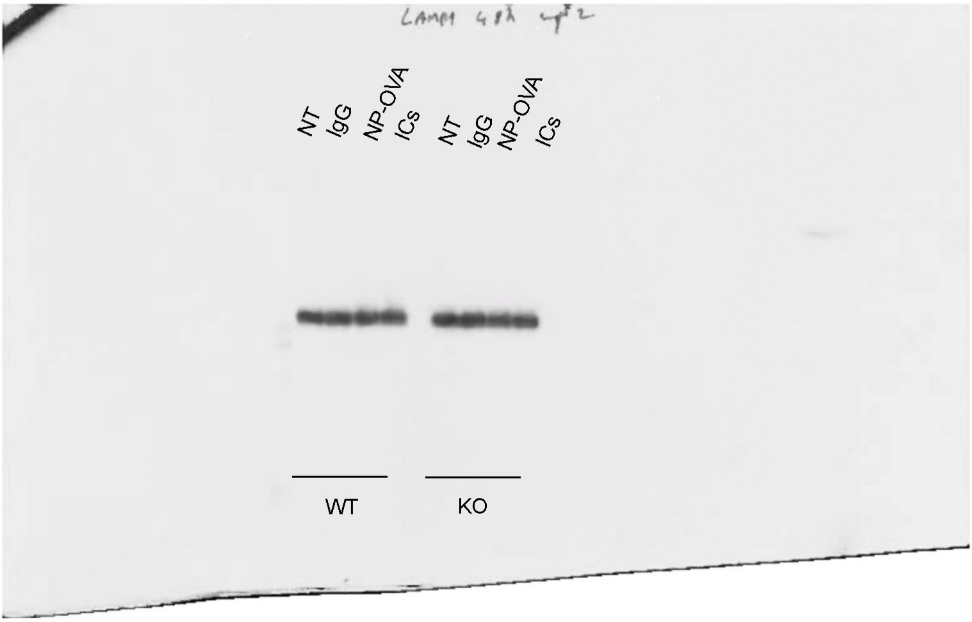

Cathepsin B 48 h

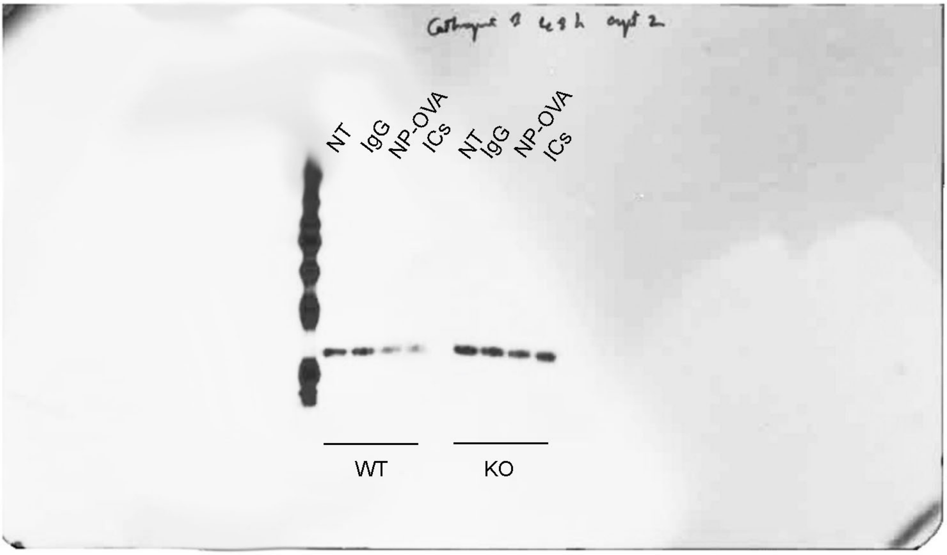

Beta actin 48 h

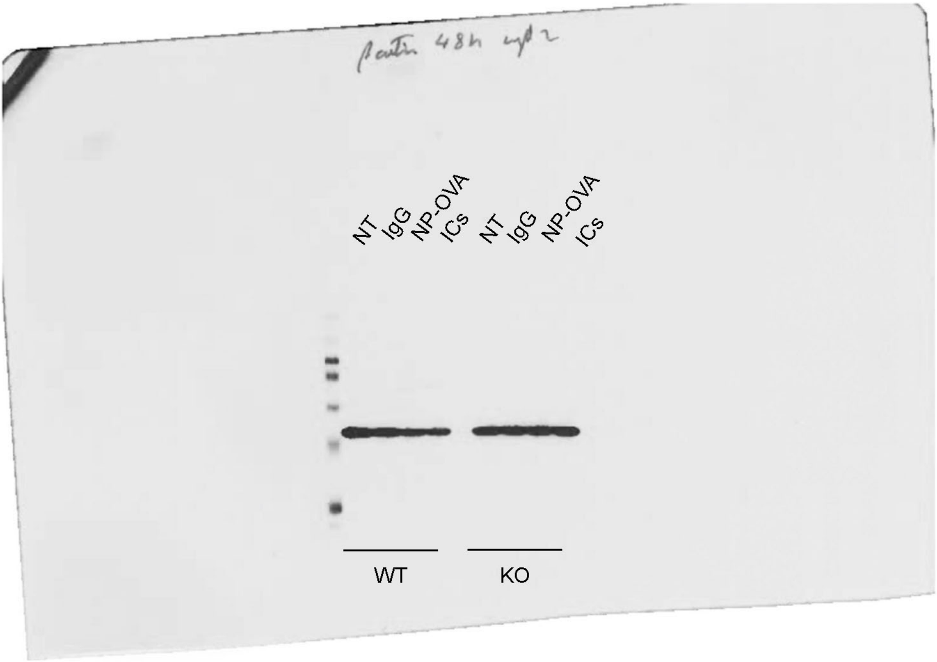

Figure 6

OVA pAkt

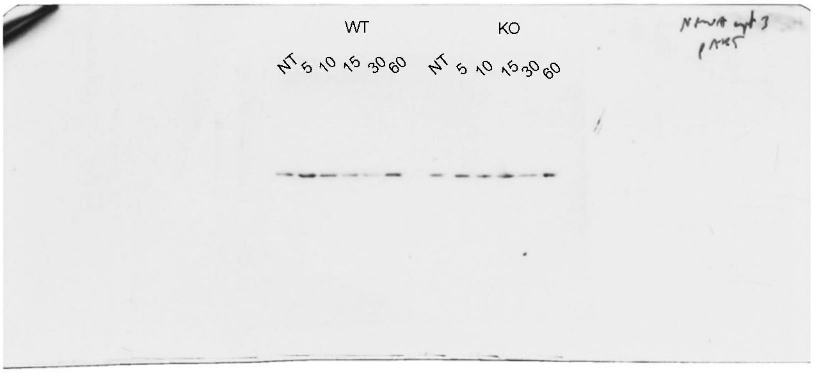

OVA Akt

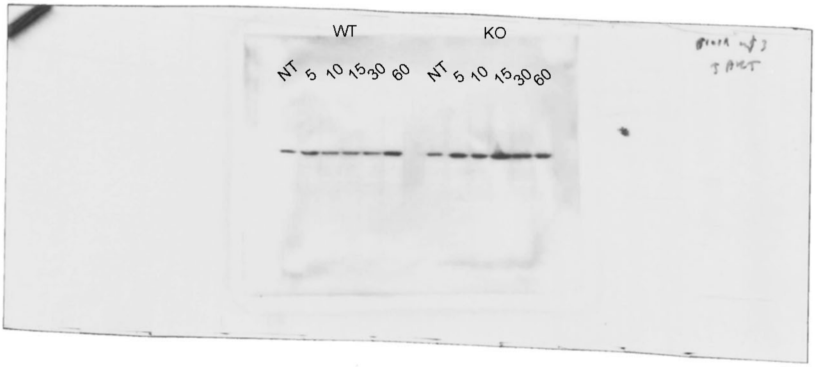

OVA pERK1/2

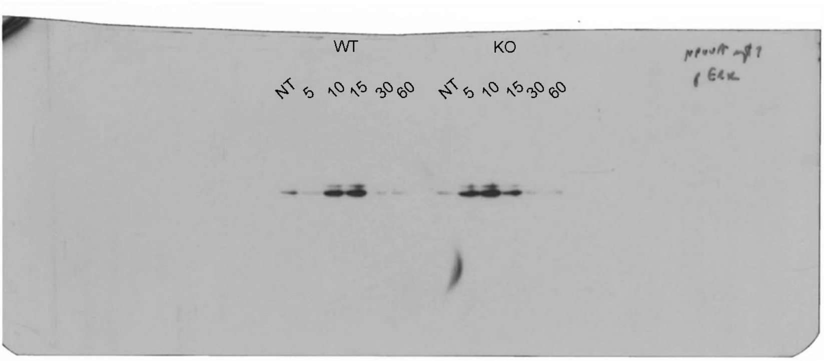

OVA ERK1

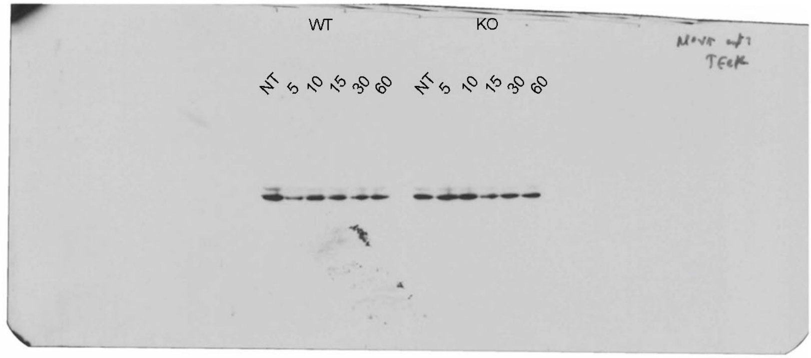

OVA actin

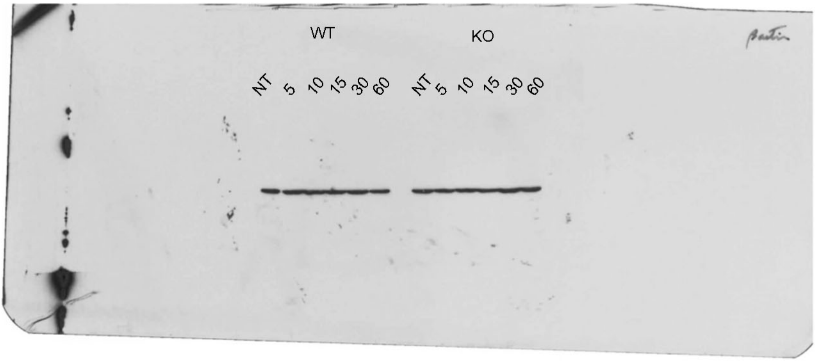

IgG pAkt

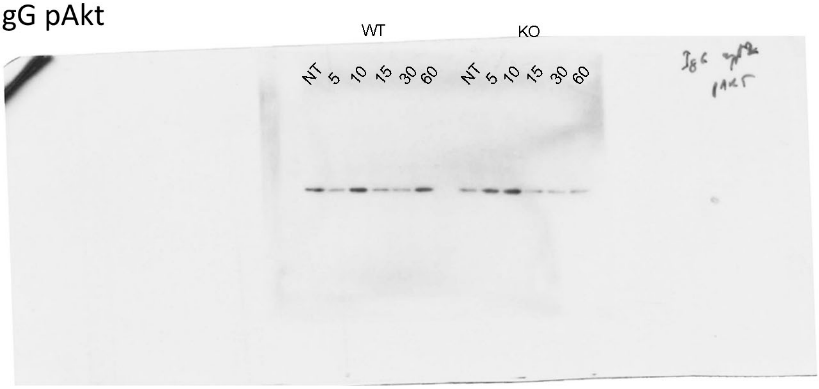

IgG Akt

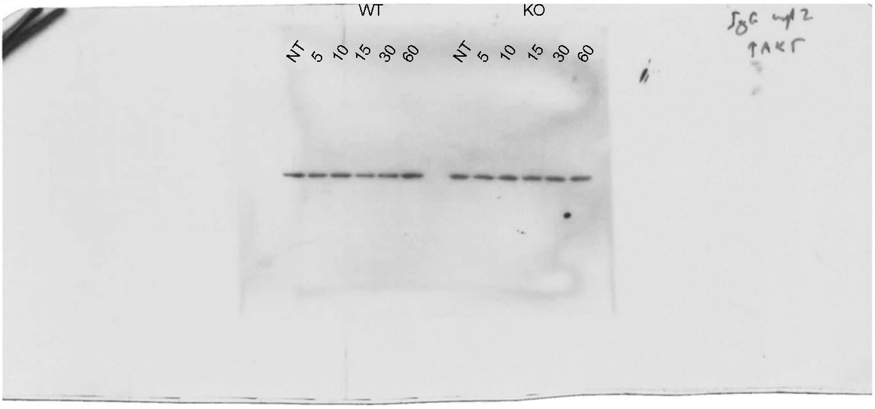

IgG pERK1/2

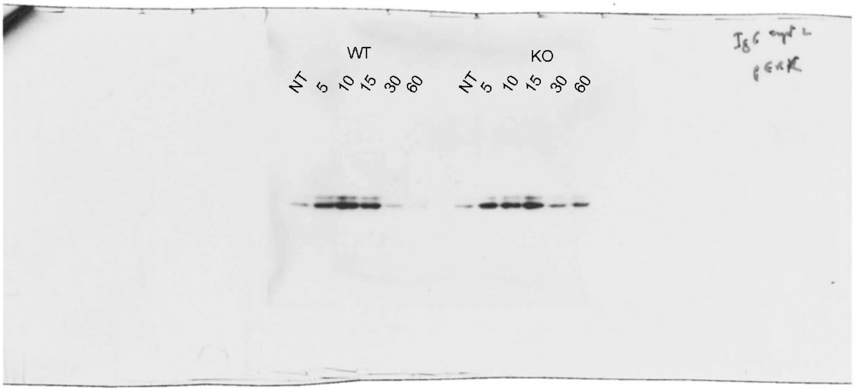

IgG ERK1

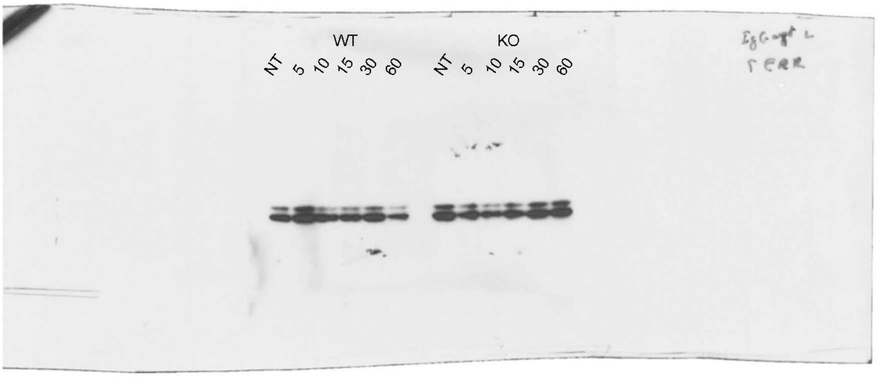

IgG actin

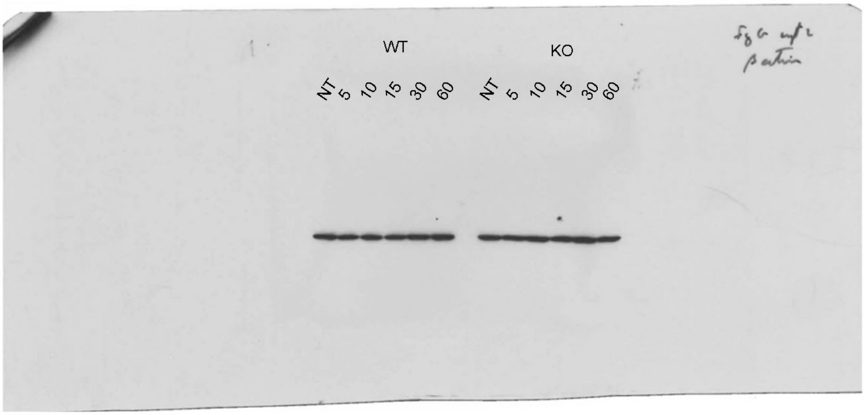

ICs pAkt

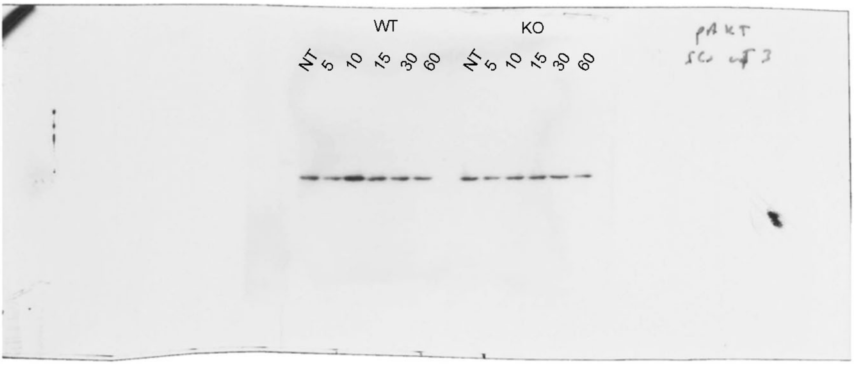

ICs Akt

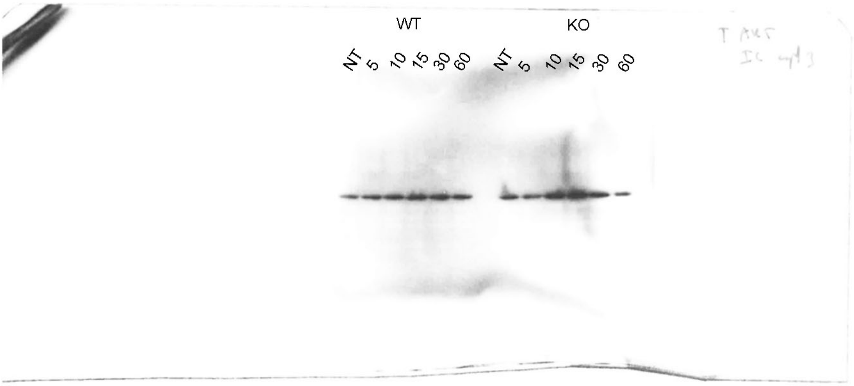

ICs pERK1/2

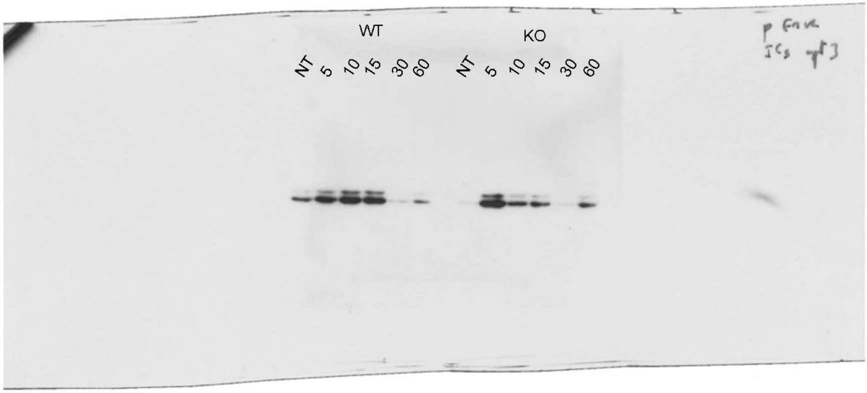

ICs ERK1

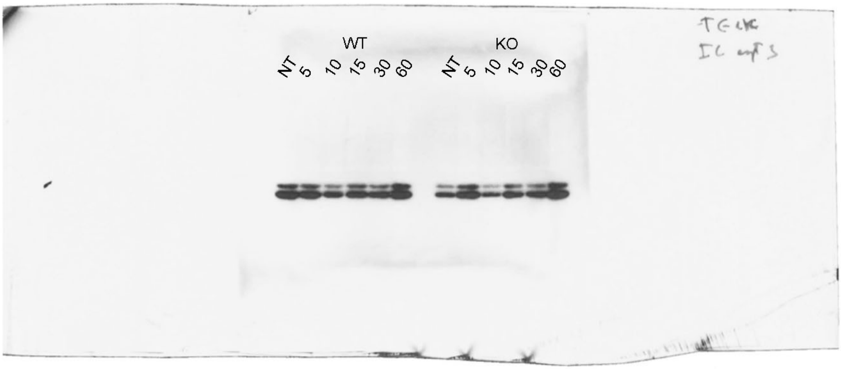

ICs actin

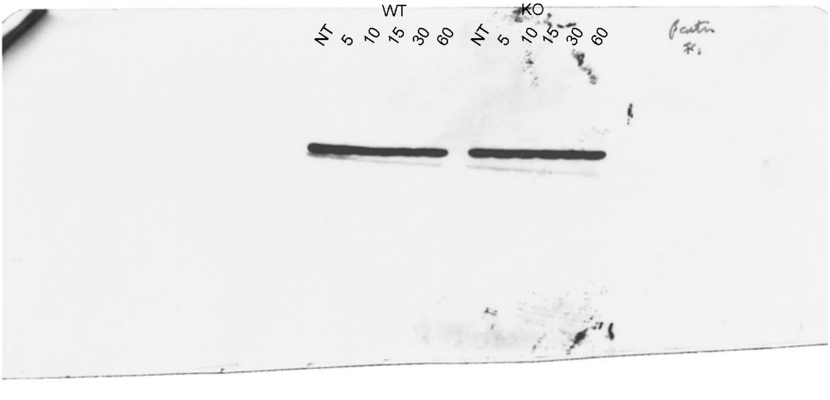

Supplement: S1 Fig — For Fig 4, uncropped gels are shown for LAMP1 at 4 hours, cathepsin B at 4 hours, LAMP1 at 48 hours, cathepsin B at 48 hours and corresponding beta-actin for each gel. For Fig 6, uncropped gels are shown for NP-ovalbumin (NP-Ova), IgG and immune complex (IC)- treated WT and KO podocytes for phospho-Akt (pAkt), total Akt, phosphor-ERK1/2, total ERK1/2 and corresponding beta-actin levels. (PDF) [file pone.0284636.s001.pdf]
