# Supplementary material for: Knockout of the neonatal Fc receptor alters immune complex trafficking and lysosomal function in cultured podocytes
Source: PLoS One. 2023 Apr 18;18(4):e0284636. doi: 10.1371/journal.pone.0284636 (PMC10112810; doi:10.1371/journal.pone.0284636)
Supplement: Attachment — Submitted filename: Response to Reviewers.docx [file pone.0284636.s002.docx]

**Response to Reviewers**

We appreciate the thoughtful and astute reviewer comments which have strengthened the manuscript. Please see our response to the reviewer’s comments below. Reviewer’s comments are italicized.

*“What I miss in the discussion is the lack of sufficient speculation about the relationship of the experiments done and the results obtained to real events in autoimmune processes and their direct impact on proteinuria. Of course, only one in vivo experiment would give a real answer to this question, but the obtained experimental data are enough to form a complete pathological picture and process occurring in immune-complex mediated diseases. Perhaps a small discussion to put together all the results obtained would give a more complete picture and the actual merits of the paper”.*

To give greater clinical context and significance to our findings we have added the following sentences in the Discussion

“Indeed, plaquenil, an agent that inhibits lysosomal function by increasing lysosomal pH [35] has long been used as a mainstay of treatment for immune-mediated diseases such as lupus and rheumatoid arthritis. An understanding of how immune complexes are trafficked to the lysosome in podocytes and how an immune insult may affect lysosomal function is of increased relevance given recent studies demonstrating that IgG or immune complex uptake by podocytes modulates immune mediated kidney diseases including lupus nephritis and membranous nephropathy [5, 6]. Furthermore, anti-nephrin autoantibodies have been found in up to one-third of patients with minimal change disease [7], suggesting that podocytes themselves are the targets of immune complexes”.

We have also added the following paragraph:

“Both the Akt and ERK1/2 pathways have been shown to be involved in signaling and lysosomal activation or antigen presentation after an immune challenge [24, 26, 36]. Our findings that KO podocytes showed little or no increase in phospho-Akt or phospho-ERK1/2 expression compared to baseline after treatment with ICs whereas WT podocytes had increased expression of these signaling mediators after IC treatment support the hypothesis that FcRn-mediated trafficking of immune complexes in podocytes modulates the podocyte response to immune stimuli and that lack of FcRn dampens the response to an immune insult resulting in less metabolically active and less immunogenic lysosomes. The role of podocyte lysosomal function in glomerular health and disease is still an active matter of investigation. How lysosomal dysfunction causes podocyte loss and proteinuria in glomerulonephritis remains unknown but several studies have found dysregulation of key lysosomal components in glomerular disease. Yamamoto-Nonaka et al. found that cathepsin D expression (a lysosomal protease) was upregulated in podocytes in patients with minimal change disease (which is mediated at least in part by immune mechanisms) but not in those with focal segmental glomerulosclerosis [37]. Cathepsin D was also found to be upregulated in membranous nephropathy, another immune-mediated kidney disease [38]. In antineutrophil cytoplasmic antigen (ANCA)- mediated vasculitis, an aggressive autoimmune disease which frequently causes crescentic glomerulonephritis, a subset of patients were found to have autoantibodies to LAMP2 [39], suggesting that lysosomes may play a role in the pathogenesis of this disease. Studies in mice have also confirmed that lysosomal dysfunction plays a pathogenic role in immune-mediated kidney diseases [40].”
